# The metabolic cross‐talk between epithelial cancer cells and stromal fibroblasts in ovarian cancer progression: Autophagy plays a role

**DOI:** 10.1002/med.21473

**Published:** 2017-09-19

**Authors:** Chanitra Thuwajit, Alessandra Ferraresi, Rossella Titone, Peti Thuwajit, Ciro Isidoro

**Affiliations:** ^1^ Department of Immunology, Faculty of Medicine Siriraj Hospital Mahidol University Bangkok Thailand; ^2^ Laboratory of Molecular Pathology and Nanobioimaging, Department of Health Sciences Università del Piemonte Orientale “A. Avogadro” Novara Italy; ^3^ Visiting Professor at Department of Immunology, Faculty of Medicine Siriraj Hospital Mahidol University Bangkok Thailand

**Keywords:** autophagy, cancer‐associated fibroblast, cytokines, ovarian cancer

## Abstract

Cancer and stromal cells, which include (cancer‐associated) fibroblasts, adipocytes, and immune cells, constitute a mixed cellular ecosystem that dynamically influences the behavior of each component, creating conditions that ultimately favor the emergence of malignant clones. Ovarian cancer cells release cytokines that recruit and activate stromal fibroblasts and immune cells, so perpetuating a state of inflammation in the stroma that hampers the immune response and facilitates cancer survival and propagation. Further, the stroma vasculature impacts the metabolism of the cells by providing or limiting the availability of oxygen and nutrients. Autophagy, a lysosomal catabolic process with homeostatic and prosurvival functions, influences the behavior of cancer cells, affecting a variety of processes such as the survival in metabolic harsh conditions, the invasive growth, the development of immune and chemo resistance, the maintenance of stem‐like properties, and dormancy. Further, autophagy is involved in the secretion and the signaling of promigratory cytokines. Cancer‐associated fibroblasts can influence the actual level of autophagy in ovarian cancer cells through the secretion of pro‐inflammatory cytokines and the release of autophagy‐derived metabolites and substrates. Interrupting the metabolic cross‐talk between cancer cells and cancer‐associated fibroblasts could be an effective therapeutic strategy to arrest the progression and prevent the relapse of ovarian cancer.

**Abbreviations**
ASMA: alpha‐Smooth Muscle Actin
CAF: cancer‐associated fibroblast
ECM: Extracellular matrix
EOC: Epithelial Ovarian Cancer
FAP: fibroblast activating protein
LPA: lysophosphatidic acid
MSC: mesenchymal stem cell

## 1 INTRODUCTION

Ovarian cancer is the seventh most common cancer in women, with the incidence of around 9.4 and 5.0 age‐standardized rate per 100,000 in developed and developing countries, respectively.^1^, ^2^ Despite the progress in drug discovery and improvement in the management, ovarian cancer remains the leading cause of death from gynecological cancer.^2^, ^3^ Worldwide, as many as 240,000 women are diagnosed with ovarian cancer, and approximately half of them die each year.^2^ Early diagnosis could give a high probability of recovery, yet it is difficult because of the unspecificity of the symptoms in the early phases of the disease. On the other hand, late stage ovarian cancers are aggressive, featuring rapid growth, dissemination, chemo/radio resistance, and recurrence.^4^ Ovarian cancer progression denotes not only the emergence of more aggressive clones, but also reflects the dynamic changes in the microenvironment, in its cellular and molecular composition, as well as in the heterotypic interactions between tumor and stromal cells.^5^ Alike other solid tumors, the ovarian cancer tissue is composed of epithelial‐derived cancer cells embedded in a tumor stroma, which consists of heterogeneous cell types and a mixture of amorphous components.^6^ The latter forms the extracellular matrix (ECM) and includes structural and specialized proteins and proteoglycans. Various cell types are found in the stroma of ovarian cancer, including immune and inflammatory cells (such as lymphocytes, macrophages, and mastocytes), endothelial cells, adipocytes, and the “cancer‐associated fibroblasts” (CAFs).^7^, ^8^ The latter definitely represent the major cell component in the activated stroma.

For long time the potential role in carcinogenesis of stromal cells has been neglected, as they were regarded just as part of an inflammatory reaction induced by necrotic cancer cells. It is now recognized that the stroma composition and architecture, in terms of vascularization, type of cells, and of their secretion, play a role in the establishment and progression of cancer cells. This fact was appreciated since the “seed and soil” paradigm of metastasis formation formulated by Stephen Paget, in which the stroma (the soil) is a determinant factor in allowing the cancer cell (the seed) to take root.^9^, ^10^ It is now well established that the stroma contributes to ovarian tumorigenesis and progression.^11^, ^12^

An important notion is that the stroma itself, in terms of cell and molecular composition and architecture, is subject to dynamic changes that parallel the growth and progression of the tumor, which suggests that the two compartments coevolve and influence each other during cancer progression.^13^

The bidirectional communication between stromal and ovarian cancer cells has an impact on the metabolism, and consequently on the behavior, of the various actors.^14^ Autophagy is one such metabolic process that occurs in both cancer and stromal cells and that is influenced by and in turn influences the microenvironment.^15^ Autophagy is a lysosome‐driven process of macromolecules and organelles degradation that plays a fundamental role in cell and tissue homeostasis.^16^ In cancer cells, autophagy is clearly deregulated and contributes to abnormal growth and development of metastasis and of immune, radio, and chemo resistance.^17^, ^18^ Autophagy plays a role in cancer stemness^19^, ^20^ and in cell reprogramming,^21^ as well as in tumor cell dormancy^22^ and in tumor invasion.^23^ In the last decade, it has become evident that autophagy in stromal cells, particularly in CAFs, adipocytes, and immune cells also has a role in the carcinogenesis process, since it is involved in the secretion of cytokines and of other soluble factors that impinge on the metabolism of epithelial cancer cells. Recent studies support the view that a “metabolic symbiosis” exists between CAFs and cancer cells. In this model, cancer cells induce a rise of autophagy in CAFs, which in turn provide the cancer cells with energetic metabolic substrates and so reducing their autophagy needs.^24^, ^25^ Thus, the metabolic cross‐talk between stromal and cancer cells reciprocally affects autophagy regulation,^26^ which might reflect in behavioral changes driving cancer relapse and metastasis as we will see later.

In this review, we present the “state of art” of the current knowledge on the role of stromal cells (particularly CAFs) and of their cytokines in the development and progression of ovarian cancer, with an emphasis on the role played by autophagy in the cross‐talk between epithelial cancer cells and CAFs. This latter aspect has not been studied extensively in ovarian cancer, yet the data available support the view that, as demonstrated in other cancer models, autophagy is differentially regulated in ovarian cancer and in CAFs. Understanding how CAFs and ovarian cancer cells reprogram each other's metabolism and, in particular, how autophagy is modulated in both these cells might shed lights on novel signaling pathways that could be targeted for the treatment of ovarian cancer.

## 2 THE PROGNOSTIC IMPACT OF STROMA IN OVARIAN CANCER

During progression, ovarian cancer cells do not completely dedifferentiate and still preserve some morphological characteristics reminiscent of the anatomical region of origin. On this ground, the pathologists classify ovarian cancer in four main histotypes (serous, clear cell, endometrioid, and mucinous), featuring a diverse grade of the stroma component. Recently, a classification model of ovarian carcinomas in type I and type II, based on morphological, genetic, and clinical characteristics has been proposed.^27^, ^28^, ^29^ Type I tumors are clinically indolent, and comprise three subtypes: (1) low‐grade serous carcinomas; (2) endometriosis‐related tumors that include low‐grade endometrioid, clear cell, and seromucinous carcinomas; and (3) mucinous carcinomas and malignant Brenner tumors. These tumors apparently originate as benign hyperproliferative lesions in extra‐ovary tissues, and later implant on the ovary where eventually undergo malignant transformation. Type II tumors are clinically very aggressive, and include high‐grade serous carcinomas (accounting by far for the majority of ovarian cancers), high‐grade endometrioid carcinomas, undifferentiated carcinomas, and carcinosarcomas. High‐ and low‐grade serous carcinomas originate in the fimbriated end of the fallopian tube and, subsequently, involve the ovary. However, these tumors show a very different genetic landscape and behavior: high‐grade serous ovarian carcinomas generally bear TP53 mutations and BRCA1/2 epi‐mutations and are chemosensitive, while low‐grade serous ovarian carcinomas generally bear Ki‐RAS and B‐RAF mutations and are chemoresistant.^28^, ^29^, ^30^

Epithelial ovarian cancer cells cohabit with a variety of stromal cells embedded in the ECM to form an organoid‐like structure.^6^ In general, low‐grade serous and mucinous histotypes present with a higher content of the stroma component compared to high‐grade serous and endometrioid carcinomas.^31^ Characteristically, the clear cell histotype presents with an intense expansion of the ECM with a low infiltration of the stromal cell component.^31^

Depending on its composition, the stroma can either impede the neoplastic growth or create the conditions for cell growth and cell migration of cancer cells. Although differing from that in normal ovary tissue, the stroma in ovarian cancer may vary in the expression of genes and production of proteins that ultimately affect tumor growth and invasion.^32^ Transcriptome profiling of genes encoding signaling molecules and cognate receptors in ovarian cancer cells and matched stromal cells isolated from patients revealed the existence of two distinct stromal compartments, one permissive and one less prone to support cancer growth.^32^ Noteworthy, the former was associated with grade 3 and the latter with grade 2 ovarian serous adenocarcinomas.^32^ Consistent with the above findings, when coinjected with ovarian cancer cells in nude mice, stromal cells isolated from normal ovary were shown to restrict the tumor growth and stromal cells from cancer tissues instead promoted ovarian cancer progression.^33^

A few studies correlate the level of reactive stroma with ovarian cancer progression. It has been reported that patients bearing an ovarian carcinoma with a high content of stroma present with a high pathologic stage at diagnosis,^34^ and display a reduced overall survival and poor prognosis independently from the histotype.^35^ Along these lines, the extent of CAFs infiltration in the ovarian cancer stroma was significantly correlated with lymph node and omentum metastases and increased number of lymphatic and blood vessels, typical signs of cancer progression.^36^ Consistently, an increased content of certain ECM components produced by CAFs, such as the glycosaminoglycan hyaluronic acid and its partner glycoprotein versican, was associated with increased microvessel density, platinum resistance, and poorer overall and progression‐free survival in ovarian cancer patients.^37^, ^38^, ^39^

## 3 THE ROLE OF INFLAMED STROMA IN OVARIAN TUMORIGENESIS

The impressive and intriguing similarity in the cellular and molecular composition of the stroma formed during the healing of a wound and of the stroma surrounding epithelial cancer has been known for a long time. The wound repair by secondary intention is accompanied by a sequence of events in the stroma that include clotting, neovascularization, recruitment of macrophages and lymphocytes, activation of myofibroblasts, and release of pro‐inflammatory cytokines (e.g., TNF‐α, TGF‐β, IL‐6, and IL‐1β). This sequence of events causes an intense remodeling of the ECM, with dynamic alternation of degradation and synthesis of fibrous proteins, eventually leading to the formation of a scar. Strikingly, a similar scenario occurs during the evolution of infiltrative epithelial tumors, with the exception that cancer cell proliferation and necrosis maintain a pro‐inflammatory environment that eventually results in a desmoplastic stroma.^40^ This analogy has suggested to Dvorak the paradigm that “cancer is a wound that never heals.”^40^, ^41^ This paradigm perfectly fits with the inflammatory process that associates with ovarian cancer development and progression.^42^, ^43^, ^44^ At each ovulation, the follicle wall ruptures to release the ovum and is thereafter repaired through controlled inflammatory events much alike the wound healing.^45^ During this process, macrophages and fibroblasts are recruited to the wounded epithelial surface, and an enormous amount of cytokines/chemokines and matrix‐remodeling enzymes (including prostaglandins, bioactive eicosanoids, plasminogen activators, collagenases, interleukins,^33^ tumor necrosis factor α [TNF‐α], and various growth factors) are released on site.^46^, ^47^, ^48^ Therefore, the ovarian surface epithelial cells adjacent to the site of ovulation are exposed to an inflammatory and oxidative environment that enhances the risk of malignant transformation.^49^, ^50^ In addition, the chemokines and cytokines released in this context attract and promote the adhesion of extra‐ovarian malignant cells to the ovary.^51^ Thus, the enhanced risk of ovarian carcinogenesis associated with repetitive ovulation would arise from the continuous creation of an inflammatory microenvironment that favors either the local malignant transformation or the homing of extra‐ovarian malignant cells and, thereafter, cancer progression. Conceivably, factors other than inflammation, for instance the balance between estrogens and progesterons, are also involved in ovarian tumorigenesis.^52^, ^53^ Yet, it is a fact that inflammation in the peritoneal cavity determines a stromal environment permissive for ovarian cancer progression.^54^, ^55^, ^56^, ^57^

## 4 OVARIAN CANCER CELLS PROMOTE THE RECRUITMENT AND ACTIVATION OF STROMAL FIBROBLASTS

Active CAFs are marked by the characteristic expression of alpha‐smooth muscle actin (ASMA) and fibroblast‐activated protein (FAP).^58^ Studies conducted in a multitude of carcinomas have shown that CAFs may originate from different sources.^59^ CAFs have been reported to derive from: (1) the conversion of fibroblasts locally present in the ECM,^60^ (2) the differentiation of bone marrow derived precursor cells,^61^ (3) the trans‐differentiation of malignant epithelial cells (through EMT, epithelial‐mesenchymal transition)^62^ or of endothelial cells (through EndMT, endothelial‐mesenchymal transition),^63^ and (4) the differentiation of mesenchymal stem cells (MSCs).^64^, ^65^

**Figure 1:**
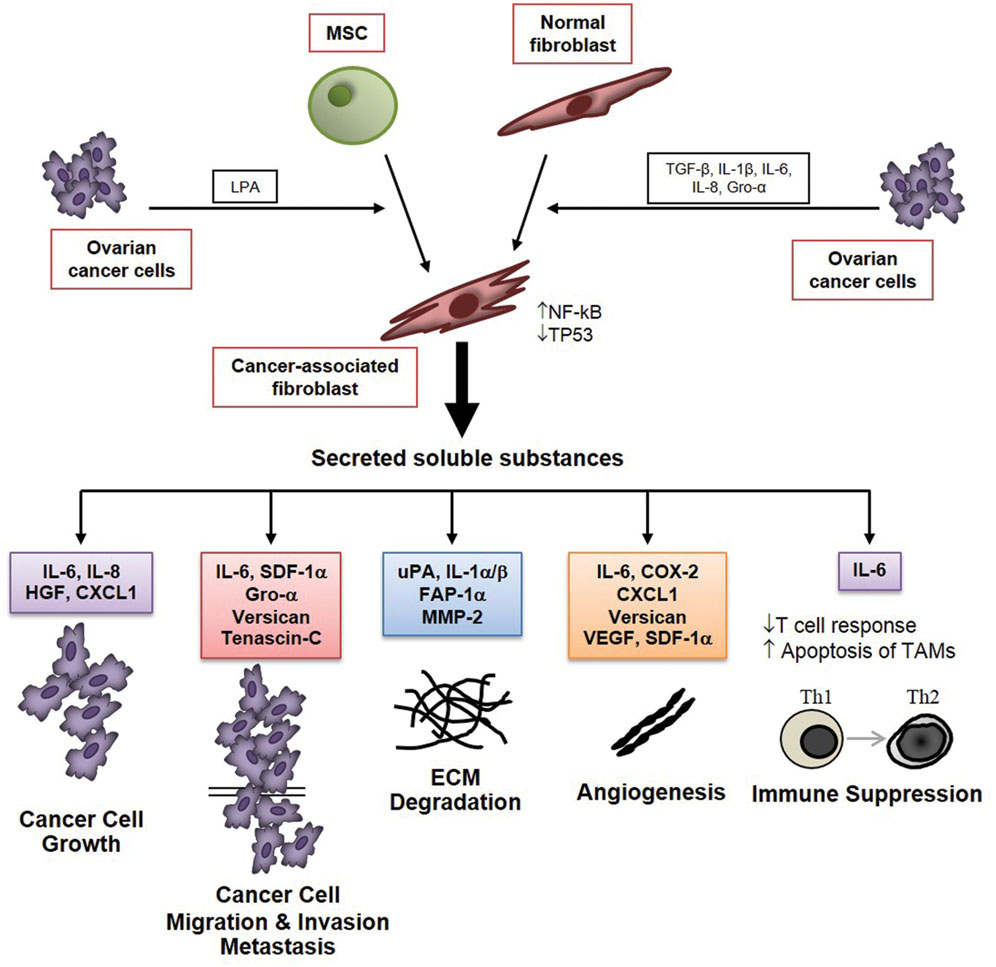
Origin and tumorigenic roles of ovarian cancer associated fibroblasts *Note*: Ovarian cancer cells secrete cytokines and soluble factors that induce the recruitment, differentiation, and activation of cancer‐associated fibroblasts (CAFs). The latter secrete a set of soluble factors, including extracellular matrix components and cytokines, that affect the behavior and the fate of ovarian cancer cells (for abbreviations, see the text). The cytokines released in the cancer stroma elicit both paracrine and autocrine stimulations on cancer cells, fibroblasts, immune cells, and endothelial cells, thus amplifying and perpetuating the effects.

Ovarian cancer cells release chemotactic cytokines and growth factors that recruit and activate the cells in the stroma (Fig. 1). The conditioned medium of ovarian (SKOV3) cancer cells, as well as TGF‐β1, could induce the trans‐differentiation of stromal fibroblasts into ASMA‐expressing myofibroblasts.^66^ The conversion of adipose‐derived and bone marrow derived MSCs and of peritoneal fibroblasts into CAFs was observed in a xenograft transplant of human ovarian cancer SKOV3 cells transgenically expressing the homeobox gene HOXA9.^67^ This effect was mediated by the release into the peritoneum of TGF‐β2 driven by HOXA9 from the ovarian cancer cells.^67^ Interestingly, the concomitant transplantation of ovarian cancer cells expressing transgenic green fluorescent protein could exclude the conversion of these cells into CAFs, which suggests that generation of CAFs through EMT of epithelial ovarian cancer cells is a rare event.^67^ The conversion of adipose‐derived MSC into CAFs within ovarian cancer stroma can be induced also by lysophosphatidic acid (LPA) released by the ovarian cancer cells.^64^ An important issue is whether the characters acquired by CAFs are stably maintained also in the absence of malignant cells, or whether these phenotypic changes are reversible. Both genetic and epigenetic factors could be involved in stabilizing the phenotype of CAFs. However, loss of heterozygosity and alterations of chromosomal copy number were found extremely rare in CAFs from ovarian cancer cells.^68^ It is therefore likely that the activated phenotype of CAFs is maintained through the epigenetic regulation of gene expression. In support of this hypothesis, a distinct pattern of DNA methylation has been reported in CAFs isolated from breast and prostate cancers.^69^, ^70^ Such a study has yet to be done in CAFs isolated from ovarian cancer. For sure, micro‐RNAs play a big role in modulating the phenotypic characters of fibroblasts in the ovarian cancer microenvironment.^71^ Differences in the expression of a subset of 11 micro‐RNAs have been reported between normal omental fibroblasts and CAFs from omental tumors, with miR‐31 and miR‐214 being the most downregulated and miR‐155 being the most upregulated in the latter.^72^ Interestingly, playing with the transfection of specific miRNAs and anti‐miRNAs to mimic this deregulation, it was possible to induce a functional conversion of normal fibroblasts into CAFs and vice versa.^72^

## 5 CANCER‐ASSOCIATED FIBROBLASTS SUPPORT OVARIAN CANCER PROGRESSION

The contribution of CAFs in cancer progression has been widely discussed in many different cancer models,^73^, ^74^, ^75^, ^76^ including ovarian cancer.^5^, ^8^, ^31^, ^77^ Here, we will briefly review the principal ways through which CAFs promote ovarian cancer progression (see also Fig. 1).

Ovarian CAFs express and secrete, among others, high levels of IL‐6, cyclo‐oxygenase 2 (COX‐2), and of chemokine (CXC motif) ligand CXCL‐1.^31^, ^78^ The latter promotes cell proliferation by binding to its receptor CXCR2, which is highly expressed on ovarian cancer cells.^79^ Ovarian CAFs could facilitate cancer cell spreading and invasion by releasing the hepatocyte growth factor (HGF),^80^ and collagenolytic proteases such as the Matrix Metalloprotease MMP‐2^81^, ^82^ and the urokinase‐like activator of the plasminogen (uPA).^83^ The numerous cytokines and soluble factors released by CAFs reported to promote ovarian cancer progression are listed in Table 1.

**Table 1:** Cytokines and soluble factors secreted by CAFs and proposed activity in ovarian cancer

Name	Effect	Mechanism/pathway	Reference
CCL5	Induces resistance to cisplatin	STAT3 and PI3K/AKT	164
TGF‐β	Promotes cell proliferation and angiogenesis Induces EMT, invasion and metastasis Induces MMPs production	TGF‐β/SMAD	12, 165
VCAN	Promotes cell migration and invasion	NF‐κB	84
MFAP5	Enhances cell motility and invasion	FAK/CREB/troponin C	166
NPPB	A novel biomarker for ovarian cancer	NPR1‐dependent pathway (in lung cancer), not determined in ovarian cancer	167
FAP‐1α	Induces cell proliferation and invasion	Integrin, α3β1, uPAR, and pERK	168, 169
SDF‐1 (CXCL‐12)	Induces cell growth, cell motility, and angiogenesis Induces cancer invasion, dissemination in the peritoneal cavity, and metastasis	CXCR4‐dependent pathway Cross‐talk with VEGF to induce strong angiogenesis	170, 171, 172, 173
CXCL‐11	Mediates cell proliferation and migration	CXCR3‐dependent pathway	174
CXCL‐1	Induces cancer‐promoting inflammation	CXCR2‐dependent pathway	78, 173
IL‐6	Mediates cancer‐promoting inflammation	IL‐6R/JAK2/STAT3	78, 173
HGF	Induces cell migration and invasion	c‐met‐dependent pathway	175
LPA	Promotes cell proliferation, invasion, and chemoresistance	LPA2‐dependent pathway	12
MMP‐2, MMP‐9 MT1‐MMP	High level in advanced EOC correlates with poor disease‐specific survival	Degrade ECM	175, 176
MMP‐1	Activates the production of CXCL1 and CXCL8 from cancer cells	PAR1 activation	177
VEGF	Direct effect in angiogenesis Cross‐talk with MMPs and CXCL12 Induces expression of *EZX2* leading to cancer cell migration	‐ VEGFR‐2‐dependent pathway: RAS/Raf/MAPK, PLC‐γ, PI3K/AKT ‐ Synergistic angiogenic effects	171, 178, 179, 180
TNF‐α	TNF network (TNF, CXCL12, IL6) inducing angiogenesis, inflammation, and leukocyte infiltration	TNFR1‐dependent pathway and NOTCH signaling	173, 181, 182, 183

*Note*: CCL5, cisplatin‐induced chemokine (C‐C motif) ligand 5; TGF‐β, transforming growth factor β; VCAN, versican; MFAPA5, microfibrillar‐associated protein; NPPB, natriuretic peptide B; NPR1, NPPB receptor; FAP, fibroblast activation protein 1α, SDF‐1, stromal‐derived factor‐1 or CXCL‐12, CXCL‐11, CXCL‐1; IL‐6, interleukin 6; EZH2, enhancer of zeste homologue 2; LPA, lysophosphatidic acid; ECM, extracellular matrix.

The secretion of versican in the ECM is another means through which CAFs may promote ovarian cancer motility, spreading, and invasion. Under TGF‐β stimulation, CAFs release high amount of versican, along with MMP‐9, resulting in increased aggressiveness of ovarian cancer.^84^

**Figure 2:**
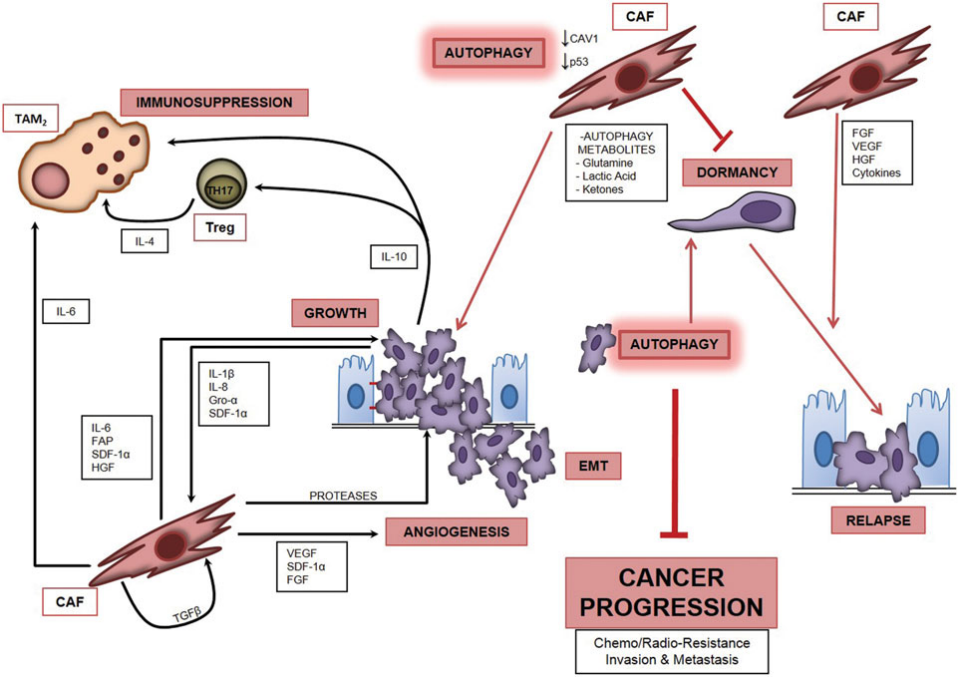
The cross‐talk between ovarian cancer cells and cancer‐associated fibroblasts in the regulation of autophagy and its role in cancer progression *Note*: Autophagy in cancer cells opposes malignant progression and promotes dormancy. Cytokines and other factors secreted by ovarian cancer cells activate autophagy in CAFs, which then secrete a vast array of soluble factors including proteases, cytokines, growth factors, and metabolites that ultimately create a microenvironment favorable to cancer growth, metastasization, and onset of chemoresistance. The inflammatory stroma could also create the conditions for awakening the dormant cancer cells, thus favoring cancer relapse (for abbreviations and a detailed description, refer to the text).

CAFs are found abundantly in the proximity of the neo‐formed blood vessels. It has been demonstrated that under LPA stimulation, the active fibroblasts express and release the CXC chemokine ligand 12/stromal cell derived factor 1α (SDF‐1α), the Vascular Endothelial Growth Factor (VEGF‐A), and IL‐6, which promote the recruitment of endothelial cells and the angiogenic sprouting in the tumor context.^85^ These events clearly favor the growth and metastasization of ovarian cancers.^86^ Finally, CAFs contribute to cancer progression also by suppressing the immune response. It has been reported that a high ratio of tumor infiltrating T‐cytotoxic versus T‐regulatory (Treg) lymphocytes associates with a better prognosis in ovarian cancer patients.^87^, ^88^ Conversely, the presence of Treg lymphocytes in the tumor microenvironment of ovarian cancer tissues creates an immunosuppressive condition that negatively affects the patient's survival.^89^, ^90^ Besides ovarian cancer cells, also the CAFs release immune‐modulatory cytokines that eventually suppress or limit the immune response^8^, ^57^ (Fig. 2).

## 6 AUTOPHAGY IN CANCER AND ITS MODULATION BY MICROENVIRONMENTAL FACTORS

Autophagy (literally, self‐eating) is a catabolic process that accomplishes the degradation within lysosomes of aged, redundant, or abnormal molecules and organelles.^91^ In this process, the autophagy material to be degraded is sequestered within the double‐membrane vesicles called autophagosomes, which eventually fuse with endosomes and lysosomes. For a more in‐depth description of the morphological and biochemical aspects of autophagy, the readers may refer to some excellent reviews.^92^, ^93^

In normal quiescent cells, autophagy runs at low basal level and allows the turnover of macromolecules and organelles/membranes without net increases in cell mass. Growth factors and nutrients (including amino acids and glucose) stimulate cell growth and proliferation and concomitantly downregulate autophagy. The progrowth signals activated by these factors converge on and positively activate mTOR.^94^ In turn, mTOR inhibits the ULK1 and BECLIN‐1–PI3kC3 complexes, thus preventing the formation of autophagosomes.^95^, ^96^ In metabolite‐ and energy‐restricted situations, the autophagy increases above the baseline in order to provide the needed substrates through the degradation of redundant constituents.^97^ In these situations, the lack of oxygen or of energy suppliers activates the AMPk pathway that switches off mTOR and activates ULK1, which then promotes the formation of the BECLIN‐1–PI3kC3 autophagy.^97^ Activation of the latter leads to the production of phosphatydilinositol‐3‐phosphate (PI3P), the biochemical signal for the recruitment of autophagosomal membranes.^95^, ^96^

Autophagy cooperates with the DNA repair systems to prevent chromosomal instability and failure in the upregulation of autophagy under conditions of genotoxic stress may lead to cell transformation.^98^ As such, autophagy acts as the guardian of both the genome and the proteome, thus accomplishing an anticancer preventive function. Yet, this same function may serve to repair the DNA damage induced by anticancer drugs, thus conferring chemo resistance to cancer cells.^99^ In a similar fashion, the upregulation of autophagy may turn of advantage for cancer cells by conferring an abnormal resistance to nutrient depletion.^100^, ^101^ It is known that the majority of cancer cells in the most inner part of fast growing and insufficiently vascularized tumors die by necrosis because of the lack of energetic nutrients and of oxygen.^102^ However, despite the lack of external nutrients those cancer clones may survive in a dormant state^103^ by raising the level of basal autophagy.^22^

Autophagy is deregulated in cancer cells,^104^, ^105^ because of mutations or epimutations of autophagy genes (e.g., *BECLIN‐1*) or autophagy‐regulating genes (e.g., *PTEN*, *TP53*). As a matter of facts, a large number of oncogenes and of oncosuppressor proteins regulate directly, or indirectly, the autophagy process.^106^ Interestingly, many autophagy genes (e.g., *BECLIN‐1*) act as oncosuppressors. The expression of autophagy genes and of autophagy‐regulating genes is modulated also at epigenetic level through histone deacetylation and promoter methylation events, as well as by certain micro‐RNAs.^107^, ^108^, ^109^, ^110^ This fact opens the possibility that environmental factors could affect the level of autophagy in a cell lineage in a chronic “stable” manner. As better illustrated below, cytokines and other soluble factors present in the stroma are capable of modulating the signaling pathways as well as the expression of autophagy‐related proteins that control autophagy. In addition, the vascularization of the stroma and the composition of the ECM (in terms of protein density) determine the availability and the diffusion of the nutrients, thus affecting the metabolic response in cancer cells as well as in stromal cells.^111^ The availability of oxygen, glucose, and certain amino acids (e.g., glutamine, arginine, and others) influences essentially three energetic metabolic pathways, that is, the glycolysis, the mitochondrial respiration, and autophagy. The mTORC1 complex, which positively regulates protein synthesis and cell growth, functions also as the master negative regulator of autophagy in response to metabolic stresses. In well vascularized area, the presence of growth factors and nutrients activates the PI3kC1‐AKT‐mTORC1 pathway, and maintains autophagy at very low basal level.^95^ Vice versa, the lack of growth factors or of amino acids reliefs the inhibitory action of the mTORC1 complex and elicits the raise of basal autophagy.^112^, ^113^ Further, the drop in the production of ATP that follows the lack of glucose or of oxygen activates the LKB‐AMPK pathway, which then inactivates the mTORC1 complex and directly activates the ULK1 complex, thus triggering autophagy.^114^

The fact that autophagy plays a pivotal role in the integrated response to all metabolic stresses highlights its role in the development of cancer cells,^101^ and points to the contribution of the tumor microenvironment in cancer progression through the regulation of this process.^15^, ^115^

## 7 AUTOPHAGY REGULATES OVARIAN CANCER CELL MIGRATION AND DORMANCY: ROLE OF STROMAL FIBROBLASTS

The first evidence that defective autophagy plays a role in ovarian tumorigenesis arose from the observation that transgenic mice hemizygous knock‐out for Beclin‐1, a main regulator of autophagy, spontaneously developed ovarian cancer, among others.^116^ A parallel study demonstrated that Beclin‐1 acts as a haploinsufficient oncosuppressor.^117^ Interestingly, monoallelic deletion of *BECLIN‐1* had previously been reported in up to 75% of human epithelial ovarian cancers.^118^ Remarkably, the expression of the autophagy‐active BECLIN‐1 protein has been proposed as a prognostic marker in human ovarian cancer.^119^, ^120^ However, these studies did not consider the role of BECLIN‐1‐dependent autophagy in the CAFs surrounding the ovarian cancer cells. In fact, the genetic monoallelic deletion of BECLIN‐1 clearly involves the whole cell populations in the body and therefore the metabolism of cells other than parenchymal ones is likely to be also affected. Besides autophagy, BECLIN‐1 is involved also in the control of receptor endocytosis and associated growth factor signaling,^121^ and its dysfunctional expression may have great impact on both the epithelial and stromal cells response to extracellular signals as well as on their reciprocal interaction.

Dysfunctional regulation of autophagy in ovarian cancer cells has been recently reviewed.^108^, ^122^, ^123^ Here, we provide an overview of the evidence supporting the involvement of CAFs and of the soluble factors present in the stroma in the regulation of autophagy and of autophagy‐related phenomena in ovarian cancer (Fig. 2).

A number of inflammatory‐related proteins abnormally present in the tumor context or in the ascitic fluid, and associated with ovarian cancer progression, could directly or indirectly affect autophagy.

Perhaps the most abundant cytokine accumulating in the plasma and ascitic fluid of ovarian cancer patients is IL‐6,^124^ a pro‐inflammatory cytokine secreted in large amount by CAFs and ovarian cancer cells. This cytokine has been shown to induce the anchorage‐independent growth and the migration and invasion of epithelial ovarian carcinoma cells.^23^, ^125^, ^126^ Very recently, we demonstrated that IL‐6 inhibits basal autophagy in ovarian cancer cells.^23^ More in detail, IL‐6 downregulates the expression of the GTPase Ras homolog ARH‐I/DIRAS3, which acts as a promoter of BECLIN‐1‐dependent autophagy and as an inhibitor of cell locomotion.^23^ The bioactive phospholipid LPA is another molecule highly secreted by ovarian cancer cells and found in the plasma and serum of the patients. LPA acts in an autocrine manner on ovarian cancer cells as well as in a paracrine manner on CAFs stimulating the secretion of VEGF, of cytokines (including IL‐6 and IL‐8), and of proinvasive soluble factors.^85^, ^127^, ^128^ LPA stimulates the EMT and ovarian cancer cell migration through activation of the Hedgehog pathway.^129^, ^130^ LPA was shown to inhibit starvation‐induced autophagy in prostate cancer cells.^131^ Very recently, we have tested the effects of LPA in ovarian cancer cell lines and found that it inhibits autophagy through induction of the Hedgehog pathway (Ferraresi et al., unpublished). Thus, the presence of LPA in the stroma can limit the autophagy compliance in ovarian cancer cell through a direct autocrine action or via indirect stimulation of IL‐6 by CAFs.

CAFs mediated regulation of autophagy impinges on another phenomenon linked to ovarian cancer progression and relapse, namely cancer cell dormancy. Cell dormancy refers to a low energetic metabolic state of the cell associated with cell quiescence. Dormant cancer cells are radio‐ and chemoresistant, and if rescued from dormancy, these cells restart to grow. Cell dormancy depends on microenvironmental conditions and is under epigenetic control.^104^ Multicellular spheroids of ovarian epithelial cancer were xenografted subcutaneously in nude mice and could remain in a state of dormancy for nearly 2 months.^132^ Dormancy was associated with scarce and imperfect neovasculature and no infiltration of stromal cells.^133^ Regrowth of dormant ovarian cancer cells was obtained upon gonadotropin stimulation, and was associated with angiogenesis and recruitment of ASMA‐positive stromal cells.^134^ Thus, exit from dormancy and tumor regrowth were marked by infiltration of myofibroblasts, which positively stabilized neoangiogenesis.^104^, ^134^ Worthy of note, dormancy of ovarian cancer cells was strictly dependent on the actual level of autophagy in the cancer cells. The group of Robert Bast found that ARH‐I (or DIRAS3) plays a pivotal role in the regulation of autophagy and dormancy in human ovarian cancer cells.^22^ ARH‐I is a maternally imprinted oncosuppressor downregulated in 60% of ovarian cancers. These authors demonstrated that reexpression of ARH‐I restores autophagy at high level in ovarian cancer cells. However, while the reexpression of ARH‐I caused cell death in cultured ovarian cancer cells, it enabled the autophagy‐dependent survival in a dormant state when these cells were xenografted in mice. This is consistent with the fact that autophagic cell death is a phenomenon mostly observed in vitro and hardly (if at all) in vivo. Of note, in cultured cells, overexpressing the transgenic ARH‐I autophagic cell death was reduced in the presence of growth factors (IGF‐1, M‐CSF), angiogenic factors (VEGF, IL‐8), and matrix proteins found in the xenografts. From these data, the authors concluded that ARH‐I can drive cancer cell dormancy in the presence of factors that promote survival in the cancer microenvironment through modulation of autophagy.^22^ From a clinical point of view, these findings suggest that relapse of ovarian cancer may result from the breakdown of dormancy induced by changes in the extent of CAFs infiltration in the tumor stroma.

## 8 AUTOPHAGY IN IMMUNE CELLS DRIVES THE SECRETION OF CYTOKINES

The secretion of proinflammatory cytokines by the immune cells in the tumor environment follows an unconventional route that exploits the vesicular traffic associated with autophagy. Deretic and colleagues showed that stimulation of autophagy enhances the secretion of IL‐1β by macrophages primed with pro‐inflammatory triggers.^135^ However, in another study the induction of autophagy in macrophages was shown to promote the autophagy‐mediated degradation of pro‐IL‐1β, thus limiting the secretion of mature IL‐1β upon stimulation with a pro‐inflammatory trigger.^136^ These contradictory results probably relied on the diverse activation of mitophagy, which dampens the mitochondrial release of reactive oxygen species. Yet, another study showed that starvation‐ or interferon‐γ‐induced autophagy in macrophages and T lymphocytes could promote the secretion of TNF‐α.^137^ Interestingly, IL‐2, a cytokine released by T lymphocytes, promotes the survival and proliferation of stromal fibroblasts through induction of autophagy.^138^

## 9 AUTOPHAGY MEDIATES THE CYTOKINE‐INDUCED DIFFERENTIATION OF FIBROBLASTS INTO MYOFIBROBLASTS

A few studies link the activity of cytokines with the autophagy process in the CAFs. Starvation‐induced differentiation of fibroblasts in myofibroblasts, as marked by the de novo synthesis of ASMA and increased expression of stress fibers, strictly depended on induction of autophagy.^139^ Similarly, one can hypothesize that the differentiation of stromal fibroblasts into myofibroblasts induced by TGF‐β1 and other cytokines secreted by ovarian cancer cells^66^ occurs through the upregulation of autophagy. In this same line, it is interesting observation that IL‐1β secreted by ovarian cancer cells attenuates the expression of p53 in neighboring CAFs,^31^ given that cytoplasmic p53 is a known downregulator of autophagy.^140^ As said before, sustained autophagy (as induced by IL‐2) is necessary for myofibroblasts survival in the stroma.^138^

## 10 METABOLIC INTERPLAY BETWEEN CAFS AND OVARIAN CANCER CELLS: AUTOPHAGY AS A DRIVING FORCE TO CANCER PROGRESSION

Autophagy is greatly influenced by an array of factors in the tumor microenvironment, such as hypoxia, pH, oxidative stress, ammonia, glucose and amino acid availability, cytokines, hormones, and growth factors.^141^, ^142^, ^143^, ^144^, ^145^, ^146^, ^147^ The physical interaction of tumor cell with surrounding cells (inflammatory cells, fibroblasts) in the matrix also influences the autophagy compliance, and consequently also the survival or death, of the tumor cell. In addition, the cytokines released by both CAFs and ovarian cancer cells have an impact on the composition of the stroma by recruiting other cells, and thus contribute to create a microenvironment that ultimately affects the regulation of autophagy in ovarian cancer cells.

It has been proposed that the functional cross‐talk between tumor cells and stromal cells through the exchange of soluble factors finally results in the reprogramming of the latter toward a metabolic state that is permissive for the growth and metastasization of the cancer cells.^148^ In this respect, autophagy may represent the target and the pivotal driver at the same time of such metabolic reprogramming.

**Figure 3:**
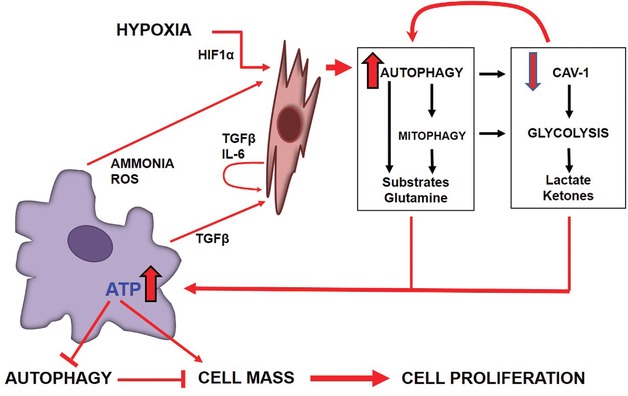
The reciprocal regulation of autophagy between cancer cells and cancer‐associated fibroblasts *Note*: The cytokine‐mediated cross‐talk and the exchange of metabolites between cancer cells and CAFs reciprocally regulate the actual level of autophagy in the cells. Cytokines, reactive oxygen species (ROS), and ammonia released by cancer cells as well as hypoxia (which triggers the HIF1α pathway) and lack of nutrients in the tumor microenvironment induce autophagy in CAFs, which leads to loss of mitochondria (mitophagy) and of Caveolin 1. The latter events favor glycolysis. Because of increased autophagy and glycolysis in CAFs, metabolites (including glutamine), lactic acid, and ketones are supplied to cancer cells. As a result, in these cells autophagy is downregulated, while anabolism is stimulated, with consequent growth of cancer.

Lisanti and colleagues have recently proposed a new paradigm of the mutual interaction between CAFs and cancer cells, in which the former supply energetic metabolites to the latter.^26^, ^149^ In such situation, autophagy in cancer cells would be maintained at low basal level, while autophagy in CAFs would be upregulated. A number of inflammatory cytokines present in the stroma and the hydrogen peroxide released by ovarian cancer cells could induce autophagy in CAFs.^150^, ^151^ As a result, CAFs would fuel the metabolism of neighboring cancer cells with autophagy metabolites such as amino acids, fatty acids, ketones, and lactate,^26^, ^149^, ^152^, ^153^ thus supporting the growth and propagation of cancer cells while keeping autophagy at a minimal level. In this model, the so‐called “Warburg effect,” that is, the aerobic glycolysis, occurs in CAFs rather than in cancer cells, which suggested the term “reverse Warburg effect.”^154^, ^155^ In a similar fashion, in pancreatic cancer the tumor‐induced autophagy in CAFs leads to the secretion of alanine, which outcompetes glutamine, in turn, to fuel the cancer cells in low‐glucose microenvironment.^25^ This same scenario is likely to occur also in the case of ovarian cancer.^156^ Thus, an integrated model of the interplay between CAFs and ovarian cancer cells cannot disregard the modulation of autophagy in both the cell types (see Fig. 3). Our unpublished data support the view that high infiltration of CAFs associates with a low level of Beclin‐1‐dependent autophagy in ovarian cancer cells. We have previously reported that the patients bearing an ovarian cancer highly expressing autophagy‐active Beclin‐1 could experience a better prognosis as compared to those bearing an autophagy‐defective cancer.^120^ Also, we have shown that ovarian cancer cell migration induced by IL‐6 occurs via downregulation of autophagy.^23^ Taken together, it is tempting to speculate that poor prognosis in ovarian cancer patients is due to the downregulation of autophagy in cancer cells operated by CAFs, as predicted in the model proposed by Lisanti and colleagues. If this interpretation is correct, to be effective the therapeutic strategies targeting autophagy should consider the different modulation of autophagy in CAFs and in cancer cells as influenced by the microenvironmental conditions.^24^

## 11 CONCLUDING REMARKS AND PERSPECTIVES

The tumor stroma is characterized by intense infiltration of reactive immune and inflammatory cells, neoformation of a nonfunctional network of blood vessels, recruitment of fibroblasts, and neodeposition of collagen and fibrin, besides other ECM proteins. In such a reactive stroma (also referred to as desmoplastic), fibroblasts, either locally resident or recruited from other anatomical sites, undergo a pronounced alterations in the phenotype and expression profile of cellular and secreted proteins. A complex stromal–epithelial interaction reciprocally influences the dynamic changes in the structure and composition of ECM and of tumor stroma, and alterations in this interaction play an important role in the development of cancer.^157^

In vivo, the actual level of autophagy in cancer cells is different in the different areas of the tumor considered, depending on the local level of nutrients, oxygen, and on the mixture of soluble factors, as provided by the vasculature and stromal composition. Given the pathophysiological role of autophagy in the regulation of cell survival and cell death in response to metabolic and genotoxic stresses, it appears clear that, by influencing the level of autophagy in the cancer cells, the stroma composition indirectly contributes to the onset of chemo resistance and dormancy in ovarian cancer cells, two conditions that negatively impact on prognosis. CAFs could release exosome in the stromal compartment to deliver metabolic substrates and micro‐RNAs to cancer cells, thus reprogramming the energetic metabolism of the latter.^158^

The reciprocal stimulation through physical contact and soluble factors between CAFs and cancer cells not only reprograms the expression of secretory factors, but also modifies in a dynamic way the metabolism in both cell types. In brief, we may assume that the stromal microenvironment acts as an epigenetic modifier of autophagy, with a fallout on the behavior of malignant cells and, consequently, the prognosis of cancer's patients.

Presently, autophagy is a target process in the therapy of cancer^159^ and of ovarian cancer in particular.^160^ With increasing recognition of the role of CAFs in carcinogenesis and progression, it is believed that targeting the metabolic cross‐talk between epithelial cancer cells and CAFs could open novel therapeutic strategies to fight ovarian cancer.^161^ Potential therapeutic strategies presently under investigation include, among others, inhibitors of the signaling pathways triggered by TGF‐β and HIF‐1α, two known inducers of autophagy in CAFs (Fig. 3).^162^, ^163^ Thus, learning how the actual level of autophagy in cancer cells and in stromal cells modulated by the tumor microenvironment may help to predict whether inhibitors or inducers of autophagy will achieve a long‐term therapeutic benefit.
